# Supplementary material for: HIVprotI: an integrated web based platform for prediction and design of HIV proteins inhibitors
Source: J Cheminform. 2018 Mar 9;10:12. doi: 10.1186/s13321-018-0266-y (PMC5845081; doi:10.1186/s13321-018-0266-y)
Supplement: Supplementary file 2 — Additional file 2. Source code of HIVProtI web server. [file 13321_2018_266_MOESM2_ESM.zip › HIVprotI_Source-code/hivprotI/weka-3-6-6/doc/weka/classifiers/trees/j48/ClassifierSplitModel.html]

ClassifierSplitModel


---


|  |  |  |
| --- | --- | --- |
| **Overview** \| **Package** \| **Class** \| **Tree** \| **Deprecated** \| **Index** \| **Help** \| **Weka's home** | |  |
| **PREV CLASS**   **NEXT CLASS** | **FRAMES**    **NO FRAMES**     **All Classes** |  |
| SUMMARY: NESTED \| FIELD \| CONSTR \| METHOD | DETAIL: FIELD \| CONSTR \| METHOD |  |


---


## weka.classifiers.trees.j48 Class ClassifierSplitModel

```
java.lang.Object
  weka.classifiers.trees.j48.ClassifierSplitModel
```

**All Implemented Interfaces:**: java.io.Serializable, java.lang.Cloneable, RevisionHandler

**Direct Known Subclasses:**: BinC45Split, C45Split, GraftSplit, NBTreeNoSplit, NBTreeSplit, NoSplit, ResidualSplit

---

``` public abstract class ClassifierSplitModel extends java.lang.Object implements java.lang.Cloneable, java.io.Serializable, RevisionHandler ```

Abstract class for classification models that can be used
recursively to split the data.

**Version:**
:   $Revision: 1.11 $

**Author:**
:   Eibe Frank (eibe@cs.waikato.ac.nz)

**See Also:**: Serialized Form

---

**Constructor Summary**

`ClassifierSplitModel()`


| **Method Summary** | |
| --- | --- |
| `abstract  void` | `buildClassifier(Instances instances)`             Builds the classifier split model for the given set of instances. |
| `boolean` | `checkModel()`             Checks if generated model is valid. |
| `double` | `classifyInstance(Instance instance)`             Classifies a given instance. |
| `double` | `classProb(int classIndex, Instance instance, int theSubset)`             Gets class probability for instance. |
| `double` | `classProbLaplace(int classIndex, Instance instance, int theSubset)`             Gets class probability for instance. |
| `java.lang.Object` | `clone()`             Allows to clone a model (shallow copy). |
| `double` | `codingCost()`             Returns coding costs of model. |
| `Distribution` | `distribution()`             Returns the distribution of class values induced by the model. |
| `java.lang.String` | `dumpLabel(int index, Instances data)`             Prints label for subset index of instances (eg class). |
| `java.lang.String` | `dumpModel(Instances data)`             Prints the split model. |
| `abstract  java.lang.String` | `leftSide(Instances data)`             Prints left side of condition satisfied by instances. |
| `int` | `numSubsets()`             Returns the number of created subsets for the split. |
| `void` | `resetDistribution(Instances data)`             Sets distribution associated with model. |
| `abstract  java.lang.String` | `rightSide(int index, Instances data)`             Prints left side of condition satisfied by instances in subset index. |
| `java.lang.String` | `sourceClass(int index, Instances data)` |
| `abstract  java.lang.String` | `sourceExpression(int index, Instances data)` |
| `Instances[]` | `split(Instances data)`             Splits the given set of instances into subsets. |
| `abstract  double[]` | `weights(Instance instance)`             Returns weights if instance is assigned to more than one subset. |
| `abstract  int` | `whichSubset(Instance instance)`             Returns index of subset instance is assigned to. |

**Methods inherited from class java.lang.Object**

`equals, getClass, hashCode, notify, notifyAll, toString, wait, wait, wait`

**Methods inherited from interface weka.core.RevisionHandler**

`getRevision`

**Constructor Detail**

### ClassifierSplitModel

```
public ClassifierSplitModel()
```


**Method Detail**

### clone

```
public java.lang.Object clone()
```

:   Allows to clone a model (shallow copy).

    :   **Overrides:**: `clone` in class `java.lang.Object`

---


### buildClassifier

```
public abstract void buildClassifier(Instances instances)
                              throws java.lang.Exception
```

:   Builds the classifier split model for the given set of instances.

    :   **Throws:**: `java.lang.Exception` - if something goes wrong

---


### checkModel

```
public final boolean checkModel()
```

:   Checks if generated model is valid.

---


### classifyInstance

```
public final double classifyInstance(Instance instance)
                              throws java.lang.Exception
```

:   Classifies a given instance.

    :   **Throws:**: `java.lang.Exception` - if something goes wrong

---


### classProb

```
public double classProb(int classIndex,
                        Instance instance,
                        int theSubset)
                 throws java.lang.Exception
```

:   Gets class probability for instance.

    :   **Throws:**: `java.lang.Exception` - if something goes wrong

---


### classProbLaplace

```
public double classProbLaplace(int classIndex,
                               Instance instance,
                               int theSubset)
                        throws java.lang.Exception
```

:   Gets class probability for instance.

    :   **Throws:**: `java.lang.Exception` - if something goes wrong

---


### codingCost

```
public double codingCost()
```

:   Returns coding costs of model. Returns 0 if not overwritten.

---


### distribution

```
public final Distribution distribution()
```

:   Returns the distribution of class values induced by the model.

---


### leftSide

```
public abstract java.lang.String leftSide(Instances data)
```

:   Prints left side of condition satisfied by instances.

    :   **Parameters:**: `data` - the data.

---


### rightSide

```
public abstract java.lang.String rightSide(int index,
                                           Instances data)
```

:   Prints left side of condition satisfied by instances in subset index.

---


### dumpLabel

```
public final java.lang.String dumpLabel(int index,
                                        Instances data)
                                 throws java.lang.Exception
```

:   Prints label for subset index of instances (eg class).

    :   **Throws:**: `java.lang.Exception` - if something goes wrong

---


### sourceClass

```
public final java.lang.String sourceClass(int index,
                                          Instances data)
                                   throws java.lang.Exception
```

:   **Throws:**: `java.lang.Exception`

---


### sourceExpression

```
public abstract java.lang.String sourceExpression(int index,
                                                  Instances data)
```

---


### dumpModel

```
public final java.lang.String dumpModel(Instances data)
                                 throws java.lang.Exception
```

:   Prints the split model.

    :   **Throws:**: `java.lang.Exception` - if something goes wrong

---


### numSubsets

```
public final int numSubsets()
```

:   Returns the number of created subsets for the split.

---


### resetDistribution

```
public void resetDistribution(Instances data)
                       throws java.lang.Exception
```

:   Sets distribution associated with model.

    :   **Throws:**: `java.lang.Exception`

---


### split

```
public final Instances[] split(Instances data)
                        throws java.lang.Exception
```

:   Splits the given set of instances into subsets.

    :   **Throws:**: `java.lang.Exception` - if something goes wrong

---


### weights

```
public abstract double[] weights(Instance instance)
```

:   Returns weights if instance is assigned to more than one subset.
    Returns null if instance is only assigned to one subset.

---


### whichSubset

```
public abstract int whichSubset(Instance instance)
                         throws java.lang.Exception
```

:   Returns index of subset instance is assigned to.
    Returns -1 if instance is assigned to more than one subset.

    :   **Throws:**: `java.lang.Exception` - if something goes wrong


---


|  |  |  |
| --- | --- | --- |
| **Overview** \| **Package** \| **Class** \| **Tree** \| **Deprecated** \| **Index** \| **Help** \| **Weka's home** | |  |
| **PREV CLASS**   **NEXT CLASS** | **FRAMES**    **NO FRAMES**     **All Classes** |  |
| SUMMARY: NESTED \| FIELD \| CONSTR \| METHOD | DETAIL: FIELD \| CONSTR \| METHOD |  |


---
